# Identification of canine circulating miRNAs as tumor biospecific markers using Next-Generation Sequencing and Q-RT-PCR

**DOI:** 10.1016/j.bbrep.2021.101106

**Published:** 2021-08-19

**Authors:** Payal Agarwal, Melissa P. Crepps, Natalie A. Stahr, Will P. Kretzschmar, Hannah C. Harris, Nripesh Prasad, Shawn E. Levy, Bruce F. Smith

**Affiliations:** a Scott-Ritchey Research Center, College of Veterinary Medicine, Auburn University, USA; b Department of Pathobiology, College of Veterinary Medicine, Auburn University, USA; c HudsonAlpha Discovery Life Science, Huntsville, AL 35806, USA

**Keywords:** Cancer, Circulatory miRNAs, Next-generation sequencing, Diagnostic biomarker, Dog

## Abstract

Delay in cancer diagnosis often results in metastasis and an inability to successfully treat the tumor. The use of broadly cancer-specific biomarkers at an early stage may improve cancer treatment and staging. This study has explored circulatory exosomal miRNAs as potential diagnostic biomarkers to identify cancer patients. Secretory exosomal miRNAs were isolated from 13 canine cancer cell lines (lymphoma, mast cell tumor, histiocytic cell line, osteosarcoma, melanoma, and breast tumor) and were sequenced by Next-Generation sequencing (NGS). We have identified 6 miRNAs (cfa-miR-9, -1841, −1306, −345, −132, and −26b) by NGS that were elevated in all cancer cell types. The miRNAs identified by NGS were then examined by Q-RT-PCR. The PCR data demonstrated similar expression patterns to those seen with NGS but provided fold differences that were much lower than those seen for NGS. Cfa-miR-9 was found to be the most consistently elevated miRNA in NGS and PCR, making it the most likely miRNA to prove diagnostic. In this study, we have demonstrated that it is possible to identify exosomal miRNAs with elevated secretion across multiple tumor types that could be used as circulatory diagnostic biomarkers for liquid biopsy in the future.

## Highlights

• Circulatory exosomal miRNAs are useful as diagnostic biomarkers for early cancer detection.
• Secretory miRNAs can be sequenced and identified by Next-Generation sequencing.
• Six miRNAs were elevated in canine cancer cell lines.
• Q-RT-PCR was used to analyze and validate miRNAs expression.
• Cfa-miR-9 was most consistently elevated miRNAs in all cancer cell types.

## 1 Introduction

*Cancer is the leading cause of death globally and second leading cause of death of people in the United States* [1]*.* The American Cancer Society estimates that there will be 1.9 million new cancer cases diagnosed and 600,000 cancer deaths in the United States in 2021 (1). Skin, breast, and bone cancers have clear warning signs such as swelling, enlarged lymph nodes, or bleeding. Other cancers, like liver, pancreatic, colon, ovarian, and brain cancer, are more difficult to diagnose as they occur internally and have few early signs of disease. Delay in diagnosis results is an increased risk of metastasis and reduced responsiveness to cancer treatments, which is one of the reasons behind the large number of deaths [1]. A few diagnostic markers for specific tumors are available: Prostate specific antigen (PSA) for prostate cancer, Cancer antigen 125 (CA125) for ovarian cancer, calcitonin for medullary thyroid cancer, alpha fetoprotein (AFP) for liver cancer, and human chorionic gonadotropin (HCG) for germ cell tumors. The utility of some of these markers is debatable and they are not common to all cancer types. Improved cancer detection, especially early in the disease process, and preferably utilizing non-invasive approaches, would potentially improve cancer patients’ health care substantially.

Dogs are an excellent translation model for cancer as they share the same environmental exposures and risks as humans with a high degree of similarity in gene sequences and function. Almost 50% of dogs, 10 years old or older, are diagnosed with cancer at some point in their lives [2].

Micro RNAs (miRNAs) are small, 19–25 nucleotide long, non-coding RNA molecules found in cells, that regulate post-transcriptional gene expression by inhibiting mRNA translation into protein [3]. A portion of miRNAs are shed into the circulation in lipid coated particles known as exosomes [4]. In recent years, circulatory exosomal miRNAs have been identified as possible cancer biomarkers, as they are stable in blood and are protected from endogenous RNAse activity [5]. miR-20a in prostate cancer patients [6], miR-21, -1246, and −122 in breast cancer patients [7,8], and miR-425–5p, -1180–3p, -122–5p, -24–3p, and -4632–5p in early gastric cancer [9] are some examples where circulatory miRNAs can be used as diagnostic biomarkers.

Most miRNA that have been identified as tumor markers have been found in the context of specific tumors [[10], [11], [12]]. Therefore, we aspired to identify miRNAs that more broadly recognize cancer, as opposed to specific tumor types, in order to devise a blood-based diagnostic test (aka liquid biopsy) for routine screening to diagnose early stage cancer.

## 2 Materials and methods

### 2.1 Cell culture

Canine mammary tumor cell lines CMT28 and CMT12, histiocytic cell line DH82, embryonic kidney cell lines FDK and MDCK, melanoma cell lines CML7 and CML10, osteosarcoma cell lines D17 and CF11, primary osteosarcoma cells COSC-3 and COSC-6, lymphoma cell lines 17–71 and OSW and mast cell lines MPT1 and BR and primary normal canine fibroblast (NCF) cells were cultured using standard lab techniques [13].

### 2.2 Exosome and total RNA isolation

All cells were passaged at 25% confluence and grown in complete DMEM/RPMI media with 10% exosome depleted FBS (ThermoFisher). Exosomes were isolated from media collected after 48 h using Total Exosome Isolation Reagent from cell culture media (ThermoFisher) following the manufacturer's guidelines. Total RNA was isolated using Total Exosome RNA and Protein Isolation Kit (ThermoFisher) using the manufacturer's guidelines. Isolated RNA was stored at −80 °C. 100–200 ng of the RNA was used for sequencing and 34–80 ng was used to analyze the findings of RNA sequencing by Q-RT- PCR.

### 2.3 miRNA library preparation

Libraries of small RNAs from each sample were prepared from total RNA using the NEBNext Small RNA Library Prep Set for Illumina (New England BioLabs Inc.) according to manufacturer's protocol. Briefly, 3′ adapters were ligated to total input RNA followed by hybridization of multiplex SR RT primers and ligation of multiplex 5′ SR adapters. Reverse transcription (RT) was performed using ProtoScript II RT for 1 h at 50 °C. Immediately after completion of the RT reaction, PCR amplification was performed for 15 cycles using LongAmp Taq 2X master mix. Illumina indexed primers were added to uniquely barcode each sample. Post- PCR material was purified using the QIAquick PCR purification kit (Qiagen). Post-PCR yield and concentration of the prepared libraries were assessed using a Qubit 2.0 Fluorometer (Invitrogen) and DNA 1000 chip on an Agilent 2100 Bioanalyzer (Applied Biosystems), respectively. Size selection of small RNA was done using 3% dye free agarose gel cassettes on a Pippin prep instrument (Sage Science Inc., Beverly, MA, USA). Post-size selection concentration and size estimation of the libraries were assessed using Qubit 2.0 Fluorometer and DNA High sensitivity chip on Agilent 2100 Bioanalyzer, respectively. Accurate quantification for sequencing applications was performed using the qPCR-based KAPA Biosystems Library Quantification kit (Kapa Biosystems). Each library was diluted to a final concentration of 1.25 nM and pooled in equimolar ratios prior to clustering.

### 2.4 Sequencing and data analysis

Single End (SE) sequencing (50bp) was performed to generate at least 15 million reads per sample on an Illumina HiSeq2500 sequencer (Illumina, Inc.). Post processing of the sequencing reads from miRNA-seq experiments from each sample was performed as per the Genomic Services Laboratory unique in-house pipeline. Briefly, quality control checks on raw sequence data from each sample were performed using FastQC (Babraham Bioinformatics). Raw reads were imported on a commercial data analysis platform AvadisNGS (Strand Scientifics). Adapter trimming was done to remove ligated adapters from the 3′ end of sequenced reads with only one mismatch allowed. Poorly aligned 3′ ends were also trimmed. Sequences shorter than 15 nucleotides were excluded from further analysis. Trimmed Reads with low qualities (base quality score less than 30, alignment score less than 95, mapping quality less than 40) were removed. Filtered reads were then used to extract and count the small RNA which was annotated with micro RNAs from miRBase (release 20). The quantification operation carries out measurement at both the gene level and at the active region level. Active region quantification considers only reads whose 5′ end matches the 5′ end of the mature miRNA annotation. miRNA sequences were referenced to the canine miRNA database and each miRNA expression or read count was normalized to the total read count of the samples. Fold differences were calculated for each miRNA by comparing normalized expression of miRNAs from the cancer cell line exosomes to exosomes from normal canine fibroblasts.

### 2.5 Reverse transcription and quantitative PCR

Isolated total RNA was reverse transcribed using TaqManTM Advanced miRNA cDNA synthesis kit according to the manufacturer's instructions (ThermoFisher). cDNA was non-specifically pre-amplified using the same kit before performing quantitative PCR. Targeted miRNA amplification of cfa-miR-9, -1841, −1306, −345, −132, and −26b was performed using TaqMan® Fast Advanced Mastermix and custom-ordered TaqMan Advanced miRNA Assays. All PCRs were run on a QuantStudio™ 7 Flex Real-Time PCR system.

### 2.6 PCR data analysis

ΔCt values for each sample were calculated using the mean of all miRNA ct values for that cell or cell line as the normalization control, due to the lack of an appropriate constitutively expressed control miRNA. NCF was used as a normal control. ΔΔCt values for cancer sample miRNAs was calculated relative to NCF miRNAs as a reference normal sample Finally, relative fold miRNA expression for each sample was calculated using the formula - **2^-(ΔΔCt).**

## 3 Results

### 3.1 Next-Generation sequencing and miRNA expression analysis

Primary canine fibroblasts (NCF, eleven canine cancer cell lines including; CMT12 and 28, CML7 and 10, DH82) OSW and 1771, MPT1 and BR, and D17 and CF11, and two primary canine osteosarcoma cells, COSC-3 and COSC-6 were used as *in vitro* models to isolate exosomes secreted by tumor cells (Table 1). Cells were cultured in exosome free FBS to ensure the exosomes collected from cell culture media were exclusively from the cells in culture and did not represent bovine or other species miRNA derived from the serum supplement. Secretory exosomal total RNA was sequenced by Next-Generation sequencing for small RNA sequences. miRNA sequences were referenced to the canine miRNA database and each miRNA expression or read count was normalized to the total read count of the samples. Fold differences were calculated for each miRNA by comparing normalized expression of miRNAs in the cancer cell lines to normal canine fibroblasts cells.

**Table 1:** All canine normal and cancer cells and cell lines used in this study.

Primary Cells/Cell Lines	Details	Cancer/Non-Cancer	Comments
**NCF**	Normal Canine Fibroblast	Non-Cancer	Adherent
**CML7**	Canine Melanoma Cell line	Cancer	Adherent
**CML10**	Canine Melanoma Cell line	Cancer	Adherent
**CMT 28**	Canine Mammary Tumor Cell line	Cancer	Adherent
**CMT 12**	Canine Mammary Tumor Cell line	Cancer	Adherent
**DH82**	Canine Histiocytic Cell line 82	Cancer	Adherent
**OSW**	Canine Lymphoma Cell line	Cancer	Non-Adherent
**17–71**	Canine Lymphoma Cell Line	Cancer	Adherent
**MPT-1**	Canine Mast Cell Tumor Cell Line	Cancer	Non-Adherent
**BR**	Canine Mast Cell Tumor Cell Line	Cancer	Non-Adherent
**COSC-3**	Canine Primary Osteosarcoma Cells	Cancer	Adherent
**COSC-6**	Canine Primary Osteosarcoma Cells	Cancer	Adherent
**CF11**	Canine Osteosarcoma Cell Line	Cancer	Adherent
**D17**	Canine Osteosarcoma Cell Line	Cancer	Adherent

**Fig. 1:**
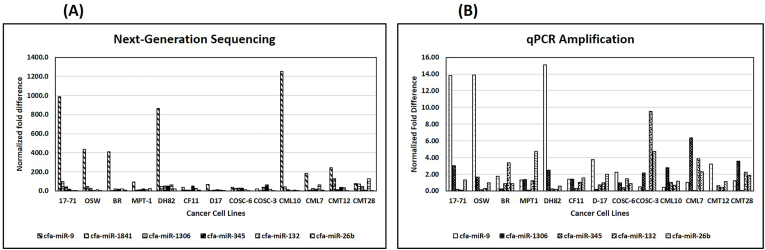
A chart representation of cfa-miR-9, -1841, −1306, −345, −132, and 26b expression fold difference in all 13 canine cancer cell lines in comparison to NCF evaluated by NGS (A) and Q-RT-PCR (B).

The top six miRNAs that were most consistently upregulated across all cancer cell lines were selected for further analysis (Table 2; Supplementary data 1). These were canine miRNAs cfa-miR-9, -1841, −1306, −345, −132, and 26b (Table 2, Fig. 1 (A)). Cfa-mir-9 was the most consistently and most highly elevated miRNA identified with levels ranging from a low of 19-fold in COSC-3 to the highest level of 1254-fold in CML10. Ten of the 13 cell lines exceeded 50-fold enhancement of miRNA expression with cfa-miR-9. cfa-miR-1841, -345, and −132 were the next most consistent miRNAs with levels ranging from 2.4-fold to 129-fold elevated expression with three cell lines exceeding 50-fold expression for each miRNA. Cfa-miR-1306 exceeded 50-fold enhancement in only one cell type (DH82). cfa-miR-26b was one of the lowest elevated miRNAs expressed across all cell lines with none of the cell lines expressing more than 50-fold over NCF.

**Table 2:** Next-Generation sequencing data of all 6 selected miRNAs in 13 canine cancer cell lines. The values depicted here are the miRNA expression fold difference in comparison to NCF. All the values in Bold highlights fold differences that exceed 50-fold.

	17–71	OSW	BR	MPT-1	DH82	CF11	D17	COSC-6	COSC-3	CML10	CML7	CMT12	CMT28
**cfa-miR-9**	**986**	**435**	**412**	**95**	**862**	38	**66**	39	19	**1254**	**183**	**242**	**77**
**cfa-miR-1841**	**96**	46	2.4	8.1	46	6.4	3.7	27	6.0	41	10	**129**	**72**
**cfa-miR-1306**	41	26	21	14	**51**	6.7	8.4	28	37	11	25	13	45
**cfa-miR-345**	16	5.2	16	23	**50**	**51**	13	28	**63**	4.3	16	38	6.8
**cfa-miR-132**	3.3	12	22	12	**65**	23	10	14	18	9	**63**	36	**129**
**cfa-miR-26b**	4.5	1.3	6.3	24	21	8.9	1.8	2.7	5.8	2.3	10	2.7	4.3

COSC-6 cells showed no miRNAs exceeding 50-fold enhancement. Of the remaining 12 cell lines, 7 cell lines (OSW, BR, MPT-1, CF11, D17, COSC-3, and CML10) exceeded 50-fold enhancement of expression with a single miRNA, 3 cell lines (17–71, CML7, and CMT12) exceeded that threshold with two miRNAs, and the remaining 2 cell lines (DH82 and CMT28) exceeded that threshold with 3 or more miRNAs.

### 3.2 Relative Q-RT-PCR quantification

Total RNA samples that were used for sequencing were used for miRNA quantification using customized miRNA Q-RT-PCR assays. In order to assure that normalization of miRNAs was comparable between sequencing and PCR data, the mean of the Ct values for all miRNAs in a cell or cell line was used as the normalization control for miRNAs in that line (Table 3 and Fig. 1 (B)). Fold differences (ΔΔCt) were calculated by comparing the normalized expression in each cell line to normal fibroblasts.

**Table 3:** Relative quantification by Q-RT-PCR all 5 miRNAs (Cfa-miR-9, -345, −1306, −132, and −26b) in all 13 canine cancer cell lines. The values depicted here are ΔΔCt values in comparison to NCF. All the values in Bold highlights differences that exceeds 3-fold.

	17–71	OSW	BR	MPT1	DH82	CF11	D-17	COSC-6	COSC-3	CML10	CML7	CMT12	CMT28
**cfa-miR-9**	**14**	**14**	1.8	1.3	**15**	1.4	**3.8**	2.3	0.5	0.5	1.0	**3.2**	1.2
**cfa-miR-1306**	**3.0**	1.6	0.2	1.3	2.5	1.4	0.2	1.0	2.1	2.8	**6.4**	NA	**3.5**
**cfa-miR-345**	0.2	0.2	0.9	0.1	0.2	0.3	0.7	0.4	0.0	1.0	0.0	0.6	0.1
**cfa-miR-132**	0.1	0.3	**3.4**	1.2	0.2	1.0	1.0	1.5	**9.5**	0.7	**3.9**	0.4	2.2
**cfa-miR-26b**	1.3	1.0	0.9	**4.7**	0.6	1.6	2.0	0.9	**4.7**	1.2	2.3	1.1	1.9

Elevated miRNA expression as measured by Q-RT-PCR were lower than those measured by NGS (Table 3). Only 13 instances of 65 possible miRNA-cell line pairing tested exceeded a cut-off of 3-fold enhancement of miRNA expression. Three cell lines, CF11, COSC-6, and CML 10 showed no miRNAs exceeding the 3-fold threshold. Of the remaining 10 cell types, 7 only exceeded the threshold with a single miRNA and the remaining 3 cell types only exceeded the threshold with two miRNAs.

Cfa-miR-9 showed the most consistently elevated expression pattern, however, only 5 (17–71, OSW, DH82, D17, and CMT12) of the 13 cell types showed elevations above 3-fold increased expression (Table 3), with the highest expression level being 15-fold. Cfa-miR-1306 was elevated in 3 cell types, 17–71, CML7, and CMT28 as was cfa-miR-132, which was elevated in BR, COSC-3, and CML7. Only two cell types showed elevated levels of cfa-miR26b, MPT1 and COSC-3. The miRNA cfa-miR-345 did not show levels of expression enhancement above 1.0 in any of the cells tested.

Nearly all cells showed Q-RT-PCR amplification at some level with each miRNA. However, miR-1841 showed amplification only in CML10, 17–71, OSW, D17, and DH82 cells (Table 4). There was no amplification of miR-1841 in NCF, BR, MPT-1, CF11, COSC-3, COSC-6, CM7, CMT12, and CMT28. Since miR1841 did not amplify in NCF, relative fold difference for miR-1841were not calculated. Interestingly, using a lower threshold of 30 for NGS, 4 out of 5 cell lines, 17–71, OSW, DH81, and CML10 showed elevated expression both in NGS and Q-RT-PCR assays (Table 4).

**Table 4:** Comparison of cfa-miR-1841 expression by NGS and Q-RT-PCR. The values in the NGS data is the expression fold difference in comparison to NCF. The Q-RT-PCR values depicted are the Ct values from PCR amplification.

		17–71	OSW	BR	MPT-1	DH82	CF11	D17	COSC6	COSC3	CML10	CML7	CMT12	CMT28
**NGS**	**cfa-miR-1841**	**96**	**46**	**2.4**	**8.1**	**46**	**6.4**	**3.7**	**27**	**6.0**	**41**	**10**	**129**	**72**
**qPCR**	**cfa-miR-1841**	**27.3**	**29.9**	**NA**	**NA**	**26.3**	**NA**	**32**	**NA**	**NA**	**27.9**	**NA**	**NA**	**NA**

## 4 Discussion

There are currently 2654 human mature miRNA and 502 canine miRNA sequences identified in the miRNA database (GRCh38, CanFam3.1) [14]. miRNAs have functional roles in many different diseases including hepatitis, endometriosis, common parasitic and viral infections, cardiac diseases, neuromuscular disease, and cancer [[15], [16], [17]]. Circulatory miRNAs enveloped in exosomes are secreted by cells and are hypothesized to act in cell to cell communications locally or at a distance, depending upon the need.

In cancer, miRNAs can function as tumor suppressor genes or oncogenes. Circulatory miRNAs have been identified as markers in human [6,7,18,19] as well as canine cancer patients [[20], [21], [22]]. In particular, miRNAs have been identified as specific biomarkers for individual tumor types, such as transitional cell carcinoma of the bladder [[23], [24], [25]], osteosarcoma, and mammary cancer [[26], [27], [28], [29], [30], [31]]. Additionally, miRNAs are functionally important in lymphomas, mast cell tumors, melanomas, cutaneous squamous cell carcinoma, hepatocellular carcinoma, B-Cell chronic lymphocytic leukemia (CLL), splenic hemangiosarcoma, and acute lymphoblastic leukemia [[32], [33], [34], [35], [36], [37], [38], [39], [40]].

In addition to previous research in specific single tumor types, we extended our search to identify miRNAs that are more broadly diagnostic for cancer, as opposed to any specific tumor. *Such a search might recognize miRNAs that were previously excluded for “lack of specificity” due to their elevation in multiple tumors.* While different tumor types may have different tissues of origin and gene expression patterns, leading to tumor specific markers, tumors maintain many common features, such as unchecked growth and cell division. This study looked for miRNAs that are involved broadly in cancer mechanisms.

Micro RNAs may be identified through several different experimental approaches. Next Generation sequencing (NGS) was selected as a platform for this study as it is capable of identifying the complete range of miRNAs with a greater depth of sensitivity and accuracy. NGS allows an unbiased sampling of all existing miRNAs without needed to specify the miRNA of interest. This approach also allows quantitation. We isolated secretory exosomal miRNAs from multiple cancer cell lines, sequenced the RNA using Next-Generation Sequencing, and screened the known canine miRNAs. Based on the fold difference values, 6 miRNAs were identified that were upregulated by at least 2-fold in all, or all but one of the cells (Table 2). The high cost and time constraints involved in RNA NGS sequencing and analysis makes it difficult to use it as a routine diagnostic test. Therefore, we sought to replicate the data using Q-RT-PCR, which could be applied to clinical use. The same exosomal RNA samples used for NGS were used to amplify, analyze, and compare cfa-miR-9, -1841, −1306, −345, −132, and 26b.

In general, the PCR data demonstrated similar expression patterns as those seen with NGS but provided fold differences that were much lower than those seen for NGS (Table 2, Table 3, Fig. 1). Cfa-miR-9 was identified as being the most highly and consistently elevated of any miRNA in both NGS and Q-RT-PCR data, with 10 out of 13 cells exceeding 50-fold expression in NGS and 5 cell types out of 13 exceeding 3-fold expression in PCR. Cfa-miR-26b expression levels were least elevated in comparison to other miRNAs in both NGS and PCR assays with the exception of two cell types, MPT-1 and COSC-3, in PCR. Cfa-miR-132 was elevated above the thresholds in only 3 cell lines in both NGS and PCR, with a common cell type, CML7, in both. Although Cfa-miR-345 was above the threshold in three cell types in NGS and amplified by Q-RT-PCR in all cell types, Q-RT-PCR did not show any elevated expression in any cancer cell.

Osteosarcoma cell lines CF11 and D17, and primary osteosarcoma samples COSC-3, and COSC-6 were uniformly lower in their expression of these miRNAs than other cells, with the exception of miR-345 (NGS), where the expression was the same or higher in osteosarcoma than in other cell types. All 6 miRNAs were constantly expressed below threshold levels in COSC-6 into both NGS and PCR. Canine mast cell line MPT1 and melanoma cell line CML10 also had similarly low expression levels other than cfa-miR-9 in NGS and cfa-miR-26b in PCR exceeding the thresholds.

miR-1841 did not amplify in NCF, making it impossible to normalize to the NCF cells. As a consequence, it could not be reported in the PCR data in Table 3. However, the lack of amplification in normal cells and amplification in 17–71, OSW, DH82, and CML10 may provide a nearly binary indication that the presence of this miRNA might be indicative of cancer (Table 4). A comparison of miR-1841 NGS and PCR data shows correlation between elevated NGS values and the ability to amplify by PCR in 4 of the 5 lines showing PCR amplification (Table 4)at a lower threshold of 30.

NGS has been demonstrated to have superior analytical sensitivity, greater resolution, more comprehensive coverage, and high throughput. NGS has limitations with respect to cost, equipment, and turnaround time for results. Q-RT-PCR has lower resolution and scale, and can only examine a small set of variants. However, Q-RT-PCR is useful as a clinical diagnostic test because of its accessibility, rapid workflow and turnaround time. When the Q-RT-PCR and NGS data from this study were compared, Q-RT-PCR was similar, although not identical to the NGS results. However, Q-RT-PCR appeared to be far less sensitive than NGS.

We did not identify a single miRNA that was consistently elevated in all cancer cell lines, however, cfa-miR-9 was elevated above the threshold in many different cancer cell types according to both NGS and qPCR. Variability in expression patterns was also seen in other miRNAs. It is unclear if the variations noted reflect specific components of tumor type, for example sarcoma versus carcinoma, more specific sources of variability such as tissue of origin, or simply differences between cell lines. Regardless of the variation, this data indicates that it is possible to identify a pan-tumor specific miRNA using NGS and validate it using Q-RT-PCR. This study was based upon *in vitro* cancer cell culture. To identify a decisive miRNAs as a diagnostic biomarker we will expand our research to *in vivo* plasma samples from patients with and without cancer.

In conclusion, the data reported herein indicates that circulatory miRNAs, which are secreted into the exosomal fraction by cancer cells, may be useful as diagnostic markers for cancer, in a broadly specific manner.

## Funding

This project was funded by 10.13039/100007579Auburn University Research Initiative in Cancer.

## Declaration of competing interest

The authors declare that they have no known competing financial interests or personal relationships that could have appeared to influence the work reported in this paper.
