# Supplementary material for: Identification of canine circulating miRNAs as tumor biospecific markers using Next-Generation Sequencing and Q-RT-PCR
Source: Biochem Biophys Rep. 2021 Aug 19;28:101106. doi: 10.1016/j.bbrep.2021.101106 (PMC8379617; doi:10.1016/j.bbrep.2021.101106)
Supplement: Multimedia component 1 [file mmc1.pdf]

Supplementary Figure 1: Next-Generation sequencing data of all miRNAs in 13 canine cancer cell lines. The values depicted here are the miRNA expression fold difference in comparison to NCF. miRNAs highlighted were selected for further screening.

	17-71		OSW		BR		MPT-1		DH82		CF11		D17		COSC-6		COSC-3		CML10		CML7		CMT12		CMT28		
Accession	FC ([17-71] vs [Normal-NCF])	Regulation ([17-71] vs [Normal-NCF])	FC ([OSW] vs [Normal-NCF])	Regulation ([OSW] vs [Normal-NCF])	FC ([BR] vs [Normal-NCF])	Regulation ([BR] vs [Normal-NCF])	FC ([MPT-1] vs [Normal-NCF])	Regulation ([MPT-1] vs [Normal-NCF])	FC ([DH82] vs [Normal-NCF])	Regulation ([DH82] vs [Normal-NCF])	FC ([CF11] vs [Normal-NCF])	Regulation ([CF11] vs [Normal-NCF])	FC ([D17] vs [Normal-NCF])	Regulation ([D17] vs [Normal-NCF])	FC ([JD] vs [Normal-NCF])	Regulation ([JD] vs [Normal-NCF])	FC ([KAITY] vs [Normal-NCF])	Regulation ([KAITY] vs [Normal-NCF])	FC ([CML10] vs [Normal-NCF])	Regulation ([CML10] vs [Normal-NCF])	FC ([CML7] vs [Normal-NCF])	Regulation ([CML7] vs [Normal-NCF])	FC ([CMT12] vs [Normal-NCF])	Regulation ([CMT12] vs [Normal-NCF])	FC ([CMT28] vs [Normal-NCF])	Regulation ([CMT28] vs [Normal-NCF])	Median
cfa-miR-9	985.8151	up	434.7519	up	412.3441	up	95.39037	up	861.9846	up	37.54732	up	66.05879	up	39.02249	up	19.02505	up	1253.646	up	183.415	up	242.383	up	76.5872	up	183.415
cfa-miR-9	985.8151	up	434.7519	up	412.3441	up	95.39037	up	861.9846	up	37.54732	up	66.05879	up	39.02249	up	19.02505	up	1253.646	up	183.415	up	242.383	up	76.5872	up	183.415
cfa-miR-9	985.8151	up	434.7519	up	412.3441	up	95.39037	up	861.9846	up	37.54732	up	66.05879	up	39.02249	up	19.02505	up	1253.646	up	183.415	up	242.383	up	76.5872	up	183.415
cfa-miR-96	70.02659	up	60.10215	up	-1	down	12.52969	up	149.6149	up	-1	down	9.322833	up	-1	down	-1	down	55.37519	up	78.48066	up	97.31536	up	48.77616	up	48.77616
cfa-miR-8865	15.653	up	68.68817	up	-1	down	23.49317	up	9.652572	up	22.90694	up	49.41102	up	-1	down	54.20411	up	88.03235	up	36.70869	up	76.78094	up	67.74467	up	36.70869
cfa-miR-1841	96.41713	up	46.48294	up	2.376624	up	8.105062	up	45.6822	up	6.436683	up	3.711168	up	26.6996	up	6.032332	up	40.69545	up	10.07775	up	129.3657	up	72.27243	up	26.6996
cfa-miR-1306	41.1921	up	25.75806	up	20.89613	up	14.0959	up	50.94413	up	6.737337	up	8.39055	up	28.37666	up	37.30175	up	11.35901	up	25.31634	up	13.39202	up	44.71148	up	25.31634
cfa-miR-345	15.653	up	5.151613	up	16.41839	up	22.71007	up	49.87162	up	51.20376	up	13.05197	up	28.37666	up	62.94671	up	4.259631	up	16.45562	up	38.39047	up	6.774466	up	22.71007
cfa-miR-8908d	-1.25605	down	2180.596	up	99.22405	up	18.704	up	836.7952	up	8.582244	up	5.937868	up	41.0763	up	20.41713	up	1796.252	up	32.2488	up	-1.25605	down	-1.25605	down	18.704
cfa-miR-8908d	-1.25605	down	2180.596	up	99.22405	up	18.704	up	836.7952	up	8.582244	up	5.937868	up	41.0763	up	20.41713	up	1796.252	up	32.2488	up	-1.25605	down	-1.25605	down	18.704
cfa-miR-8908d	-1.25605	down	2180.596	up	99.22405	up	18.704	up	836.7952	up	8.582244	up	5.937868	up	41.0763	up	20.41713	up	1796.252	up	32.2488	up	-1.25605	down	-1.25605	down	18.704
cfa-miR-132	3.279493	up	11.62074	up	22.28085	up	11.53413	up	64.89435	up	23.33298	up	10.02016	up	14.37671	up	17.63298	up	9.043432	up	63.48982	up	35.54003	up	129.4432	up	17.63298
cfa-miR-203	15.653	up	1.717204	up	23.135	up	-1	down	17.69638	up	3.368668	up	-1	down	18.05788	up	39.05027	up	-1	down	-1	down	2402.529	up	1.354893	up	15.653
cfa-miR-374b	140.877	up	22.32366	up	-1	down	166.0184	up	100.2795	up	3.368668	up	14.91653	up	15.47818	up	12.82248	up	-1	down	64.55666	up	3.571207	up	-1	down	14.91653
cfa-miR-30b	97.21339	up	13.73764	up	7.462903	up	75.17813	up	107.2508	up	11.45347	up	7.458267	up	-1	down	10.49112	up	52.53544	up	92.40464	up	13.39202	up	66.38976	up	13.73764
cfa-miR-146a	158.2765	up	53.7459	up	383.9113	up	7.702406	up	1487.616	up	-3.89074	down	-1.53975	down	25.92941	up	13.45674	up	2.943825	up	353.2879	up	45.16543	up	2.11244	up	13.45674
cfa-miR-15a	44.48748	up	8.586021	up	29.10532	up	30.54112	up	45.58159	up	6.063603	up	-1	down	12.89848	up	23.89644	up	22.71802	up	39.24033	up	-1	down	9.484253	up	12.89848
cfa-miR-8908c	-1	down	2472.774	up	926.8926	up	11.74658	up	2091.927	up	-1	down	27.03621	up	74.81121	up	12.82248	up	6067.133	up	86.07556	up	1.785603	up	-1	down	12.82248
cfa-miR-19a	99.68491	up	5.151613	up	8.209193	up	42.2877	up	36.46528	up	6.737337	up	4.661417	up	-1	down	11.6568	up	2.839753	up	16.45562	up	17.85603	up	42.00169	up	11.6568
cfa-miR-32	280.1065	up	-1	down	22.38871	up	33.67354	up	43.97283	up	-1	down	11.1874	up	-1	down	9.908278	up	25.55778	up	29.11379	up	8.035214	up	2.709787	up	11.31881
cfa-miR-8908a-5p	-1	down	688.5988	up	391.8023	up	25.84248	up	1444.669	up	9.43227	up	9.322833	up	54.17362	up	9.325438	up	2672.208	up	55.69595	up	-1	down	-1	down	9.43227
cfa-miR-8908a-5p	-1	down	688.5988	up	391.8023	up	25.84248	up	1444.669	up	9.43227	up	9.322833	up	54.17362	up	9.325438	up	2672.208	up	55.69595	up	-1	down	-1	down	9.43227
cfa-miR-8908a-5p	-1	down	688.5988	up	391.8023	up	25.84248	up	1444.669	up	9.43227	up	9.322833	up	54.17362	up	9.325438	up	2672.208	up	55.69595	up	-1	down	-1	down	9.43227
cfa-miR-184	-1.03015	down	24.49924	up	-31.4013	down	-31.4013	down	1.229577	up	2.424484	up	17.87298	up	47.56636	up	-2.24485	down	17.86078	up	13.90729	up	38.98029	up	2.329976	up	7.955528
cfa-miR-182	21.62075	up	9.541533	up	-2.5579	down	-1.13497	down	6.567114	up	5.88912	up	1.11335	up	-2.14087	down	-7.66241	down	18.6403	up	4.241052	up	66.55361	up	13.83198	up	6.567114
cfa-miR-382	1.647685	up	-1	down	-1	down	7.831057	up	15.55137	up	6.737337	up	-1	down	28.37666	up	14.571	up	53.95531	up	6.329084	up	-1	down	2.709787	up	6.329084
cfa-miR-1839	43.71161	up	10.03821	up	2.425459	up	22.60707	up	6.035596	up	2.667256	up	-1.2294	down	1.462992	up	5.390325	up	8.6563	up	1.573786	up	8.958035	up	1.123023	up	6.035596
cfa-miR-26b	4.475055	up	1.332534	up	6.310085	up	23.64438	up	21.37925	up	8.901361	up	1.841491	up	2.677759	up	5.84437	up	2.21793	up	9.848128	up	2.744229	up	4.314772	up	5.84437
cfa-miR-503	1.66956	up	24.7329	up	2.32261	up	5.129428	up	9.431414	up	9.508738	up	1.551943	up	-27.6331	down	-1.35461	down	9.557266	up	22.94979	up	5.460239	up	5.785716	up	5.129428
cfa-miR-1296	5.766895	up	-1	down	-1	down	3.915528	up	23.05892	up	-1	down	-1	down	30.95635	up	4.662719	up	1.419877	up	5.063268	up	10.71362	up	24.38808	up	5.063268
cfa-miR-15b	11.41264	up	8.749729	up	7.248704	up	12.9681	up	44.74294	up	1.609171	up	1.039127	up	-6.28026	down	3.248179	up	4.747802	up	11.08553	up	3.411842	up	2.373124	up	4.747802
cfa-miR-181d	-7.17471	down	3.315327	up	-1.24671	down	10.14692	up	45.55945	up	1.562237	up	1.122629	up	-1.34317	down	4.605455	up	6.740184	up	17.37153	up	24.06948	up	6.701378	up	4.605455
cfa-miR-221	2.597811	up	-1.82638	down	-1.10307	down	12.62864	up	32.02338	up	5.185987	up	2.850397	up	-1.80152	down	7.676374	up	5.327184	up	21.21032	up	4.570431	up	1.266868	up	4.570431
cfa-miR-1842	11.53379	up	5.151613	up	-1	down	12.52969	up	5.898794	up	-1	down	2.79685	up	-1	down	-1	down	7.099383	up	10.12654	up	4.464008	up	-1	down	4.464008
cfa-miR-103	5.417618	up	-1.27252	down	5.614072	up	27.92343	up	8.760599	up	4.506523	up	2.191634	up	-1.62727	down	4.03008	up	1.780204	up	7.102039	up	3.223787	up	1.341996	up	4.422323
cfa-miR-590	2.471527	up	-1	down	8.955483	up	4.698634	up	7.507556	up	8.084804	up	3.729133	up	-1	down	1.74852	up	4.259631	up	7.594901	up	-1	down	18.9685	up	4.259631
cfa-miR-103	5.007445	up	-1.43325	down	5.105643	up	25.8717	up	8.029351	up	4.228234	up	2.098729	up	-1.44082	down	3.792209	up	1.839871	up	6.449759	up	3.014773	up	1.257235	up	4.116924
cfa-miR-23a	1.031187	up	-1.56935	down	2.285693	up	4.008619	up	47.09668	up	3.076549	up	1.203064	up	-1.01071	down	6.212294	up	7.854027	up	13.03272	up	5.58751	up	7.929801	up	4.008619
cfa-miR-8908a-3p	-1	down	704.0535	up	100.7492	up	3.915528	up	338.9125	up	-1	down	5.5937	up	-1	down	27.97631	up	340.7703	up	22.7847	up	1.785603	up	-1	down	3.915528
cfa-miR-8908a-3p	-1	down	704.0535	up	100.7492	up	3.915528	up	338.9125	up	-1	down	5.5937	up	-1	down	27.97631	up	340.7703	up	22.7847	up	1.785603	up	-1	down	3.915528
cfa-miR-8908a-3p	-1	down	704.0535	up	100.7492	up	3.915528	up	338.9125	up	-1	down	5.5937	up	-1	down	27.97631	up	340.7703	up	22.7847	up	1.785603	up	-1	down	3.915528
cfa-miR-8908a-3p	-1	down	704.0535	up	100.7492	up	3.915528	up	338.9125	up	-1	down	5.5937	up	-1	down	27.97631	up	340.7703	up	22.7847	up	1.785603	up	-1	down	3.915528
cfa-miR-1307	3.802163	up	9.31368	up	3.342128	up	1.548924	up	16.83731	up	1.709744	up	1.832389	up	-2.22582	down	3.509194	up	2.737758	up	5.60575	up	3.887189	up	5.781119	up	3.802163
cfa-miR-107	8.690658	up	-1.95054	down	3.732042	up	5.124115	up	6.684225	up	2.614903	up	1.252519	up	-1.7312	down	4.437244	up	2.190206	up	4.094087	up	1.332751	up	1.415785	up	3.732042
cfa-miR-183	19.88555	up	17.8629	up	-4.03233	down	-1.15592	down	3.60695	up	3.087743	up	-1.56729	down	-2.7219	down	-12.6629	down	11.30429	up	2.792977	up	42.2701	up	13.38651	up	3.60695
cfa-miR-190b	37.0729	up	6.868817	up	14.92581	up	17.22832	up	21.45016	up	-1	down	-1	down	-1	down	-1	down	-1	down	13.92399	up	3.571207	up	-1	down	3.571207
cfa-miR-215	2.471527	up	1.717204	up	3.731451	up	3.915528	up	11.79759	up	-1	down	4.661417	up	-1	down	3.497039	up	18.4584	up	3.797451	up	-1	down	1.354893	up	3.497039
cfa-miR-324	3.295369	up	-1	down	10.44807	up	-1	down	25.74019	up	-1	down	2.79685	up	-1	down	-1	down	7.099383	up	44.30359	up	16.07043	up	4.06468	up	3.295369
cfa-miR-24	1.182813	up	-1.12445	down	2.675657	up	4.651676	up	5.399433	up	-1.23861	down	-1.92175	down	2.398242	up	4.650726	up	4.67006	up	2.791608	up	3.292079	up	3.127176	up	3.127176
cfa-miR-24	1.180395	up	-1.12268	down	2.673477	up	4.653054	up	5.396343	up	-1.23915	down	-1.92893	down	2.399717	up	4.65254	up	4.666468	up	2.793046	up	3.288686	up	3.123065	up	3.123065
cfa-miR-1840	5.518595	up	7.165727	up	7.560121	up	6.342158	up	3.003277	up	1.609171	up	2.277889	up	-2.01715	down	3.856213	up	3.469248	up	-1.25114	down	3.039284	up	2.194582	up	3.039284
cfa-miR-499	4.976083	up	2.35149	up	4.792856	up	5.752517	up	4.201055	up	1.280185	up	-1.01046	down	1.36921	up	2.938829	up	1.657962	up	4.393898	up	1.450033	up	4.084649	up	2.938829
cfa-miR-188	15.653	up	-1	down	4.477741	up	40.7215	up	15.55137	up	5.389869	up	-1	down	-1	down	2.914199	up	-1	down	6.329084	up	-1	down	-1	down	2.914199
cfa-miR-328	2.332084	up	1.063335	up	2.838745	up	11.08385	up	6.688672	up	-1.04868	down	2.06176	up	8.21526	up	25.46984	up	3.014478	up	2.911349	up	-11.3045	down	3.116223	up	2.911349
cfa-let-7g	1.399728	up	-11.4081	down	1.497403	up	10.15716	up	2.663958	up	4.876364	up	-1.01407	down	1.513613	up	3.785569	up	6.404529	up	2.856648	up	2.412836	up	3.748112	up	2.856648
cfa-miR-421	13.84675	up	7.29144	up	2.970779	up	24.93865	up	13.85172	up	1.847567	up	-2.02093	down	1.36921	up	1.392077	up	-7.96158	down	7.278376	up	2.843202	up	-1.39057	down	2.843202
cfa-miR-191	7.81456	up	1.374247	up	3.168316	up	8.037046	up	5.267396	up	1.880849	up	1.096963	up	1.173609	up	2.809463	up	3.018391	up	2.287779	up	3.382301	up	2.331097	up	2.809463
cfa-miR-192	3.317775	up	4.255706	up	4.385658	up	2.874739	up	6.726327	up	-2.93944	down	-6.41208	down	1.134793	up	2.802209	up	1.469119	up	1.607734	up	-9.51485	down	-1.40977	down	2.802209
cfa-miR-16	9.403947	up	1.332967	up	2.785106	up	7.146486	up	12.64799	up	1.334271	up	-2.83639	down	-1.77054	down	1.885104	up	2.388031	up	8.503102	up	2.816547	up	4.935021	up	2.785106
cfa-miR-27a	2.003116	up	1.860214	up	1.643813	up	7.237501	up	17.65385	up	-1.6724	down	1.047859	up	2.469727	up	12.18235	up	6.796745	up	9.572406	up	2.751353	up	2.834559	up	2.751353
cfa-miR-495	3.279493	up	2.73429	up	19.60715	up	6.85813	up	4.696301	up	4.291122	up	-1.25605	down	-1.25605	down	19.48907	up	1.130429	up	2.01555	up	-1.25605	down	-1.25605	down	2.73429
cfa-miR-148b	6.351478	up	1.134246	up	2.426575	up	11.23894	up	9.256583	up	2.731793	up	-1.32385	down	-1.16136	down	2.534174	up	5.005648	up	6.459124	up	1.617384	up	2.85424	up	2.731793
cfa-miR-140	4.445356	up	-1.68069	down	2.091894	up	2.748435	up	8.837268	up	2.695066	up	-1.30269	down	2.149224	up	3.628248	up	5.171229	up	4.169084	up	2.014224	up	13.31091	up	2.695066
cfa-miR-181c	2.471527	up	-1	down	-1	down	5.481739	up	33.24775	up	2.694935	up	-1	down	-1	down	11.07396	up	18.4584	up	26.58215	up	8.928017	up	1.354893	up	2.694935
cfa-miR-187	43.66364	up	8.586021	up	2.985161	up	46.98635	up	8.580064	up	-1	down	-1	down	12.89848	up	1.16568	up	19.87827	up	2.531634	up	-1	down	-1	down	2.531634
cfa-miR-30c	4.340721	up	-1.20554	down	2.510142	up	5.337993	up	4.51881	up	1.777923	up	1.726318	up	1.846125	up	3.701776	up	2.756214	up	2.094813	up	-1.13828	down	1.927103	up	2.510142

cfa-miR-30c	4.340721	up	-1.20554	down	2.510142	up	5.337993	up	4.51881	up	1.777923	up	1.726318	up	1.846125	up	3.701776	up	2.756214	up	2.094813	up	-1.13828	down	1.927103	up	2.510142
cfa-miR-16	8.785069	up	1.408574	up	2.331612	up	6.980934	up	10.89335	up	1.284086	up	-2.96403	down	-1.4607	down	2.123269	up	2.500646	up	8.321781	up	3.27399	up	5.099275	up	2.500646
cfa-miR-502	14.08253	up	-1.55433	down	-3.732	down	19.05851	up	15.57062	up	4.217502	up	2.488666	up	1.490023	up	2.192749	up	-11.2789	down	3.714936	up	3.261319	up	2.982269	up	2.488666
cfa-miR-1301	1.676185	up	-6.58306	down	14.91992	up	4.64136	up	6.404046	up	1.66877	up	-1.01046	down	-11.3045	down	2.217011	up	11.6811	up	2.351475	up	14.21601	up	7.790558	up	2.41252
cfa-miR-664	4.122791	up	-1.28004	down	2.631262	up	6.769063	up	5.184228	up	-2.61004	down	1.696534	up	-8.79236	down	2.386417	up	-1.03206	down	-2.31533	down	6.194118	up	-8.79236	down	2.386417
cfa-miR-335	-8.79236	down	-5.12016	down	6.111317	up	6.412797	up	9.819537	up	2.375443	up	1.2724	up	-8.79236	down	15.37913	up	55.39102	up	-3.473	down	1.116972	up	2.003286	up	2.375443
cfa-miR-25	5.405354	up	1.40386	up	1.979204	up	5.310339	up	9.643067	up	2.619729	up	1.401655	up	-2.01915	down	2.360724	up	2.716535	up	2.467821	up	2.150112	up	1.539796	up	2.360724
cfa-miR-99b	-261.219	down	-39.28	down	-1.51657	down	-5.23681	down	5.223506	up	1.352753	up	1.100561	up	2.262166	up	3.428992	up	7.524677	up	2.402639	up	4.721747	up	2.307288	up	2.307288
cfa-miR-93	5.133938	up	-1.08161	down	2.590349	up	4.525736	up	11.43514	up	2.29606	up	-1.52254	down	-16.9197	down	1.495564	up	2.464172	up	2.436061	up	2.290925	up	-1.19315	down	2.301478
cfa-miR-155	20.36366	up	4.17025	up	-7.48585	down	1.652813	up	4.812344	up	2.782749	up	3.579159	up	-6.90933	down	-3.02927	down	-1.12657	down	14.4707	up	2.046532	up	1.44067	up	2.249075
cfa-miR-370	-1	down	-1	down	2.238871	up	2.349317	up	-1	down	7.41107	up	2.79685	up	25.79696	up	27.97631	up	-1	down	1.265817	up	-1	down	9.484253	up	2.238871
cfa-miR-196a	11.78431	up	-37.6815	down	10.16007	up	1.41319	up	4.610913	up	1.859486	up	2.226701	up	-37.6815	down	-1.70136	down	9.985457	up	1.142145	up	1.540067	up	2.409081	up	2.226701
cfa-miR-152	-42.2626	down	-33.7931	down	2.847318	up	4.541499	up	2.105726	up	3.170353	up	-1.65544	down	3.304478	up	13.99844	up	2.321213	up	3.830418	up	-13.3556	down	2.180734	up	2.180734
cfa-miR-20a	4.833398	up	-1.66391	down	3.730422	up	8.274525	up	2.454168	up	2.16716	up	-1.08219	down	-8.06088	down	-1.34353	down	-1.74581	down	1.278318	up	2.234966	up	1.462388	up	2.16716
cfa-miR-27b	2.166545	up	-1.3997	down	-2.2613	down	1.036829	up	5.040376	up	-1.03012	down	-1.08563	down	2.192022	up	6.736218	up	4.828596	up	2.666044	up	4.821285	up	1.999412	up	2.166545
cfa-miR-340	6.612315	up	1.82286	up	2.444252	up	22.9821	up	5.661248	up	2.040901	up	-1.46651	down	-59.8885	down	3.361358	up	1.553191	up	3.38383	up	1.78567	up	-1.06567	down	2.040901
cfa-miR-23b	-1.63551	down	-3.22057	down	-1.80546	down	-1.43818	down	8.239151	up	1.469164	up	1.117125	up	1.420266	up	6.587588	up	4.678826	up	3.474261	up	4.739471	up	2.091566	up	1.970294
cfa-miR-532	14.77827	up	2.934149	up	-3.7998	down	8.732469	up	9.844418	up	1.96555	up	-1.0584	down	-1.02194	down	1.282775	up	-9.58648	down	6.683914	up	1.557271	up	2.149527	up	1.96555
cfa-miR-181b	-40.4026	down	3.443656	up	4.781273	up	1.008721	up	-2.01854	down	1.95833	up	3.37506	up	-1.22884	down	5.10647	up	10.51512	up	1.23595	up	1.736767	up	3.180109	up	1.95833
cfa-miR-181b	-40.4026	down	3.443656	up	4.831721	up	1.008721	up	-2.01854	down	1.9558	up	3.385565	up	-1.22884	down	5.099902	up	10.79772	up	1.23595	up	1.736767	up	3.180109	up	1.9558
cfa-miR-19b	23.1907	up	2.148371	up	1.103432	up	4.097065	up	4.665805	up	-2.37276	down	1.272401	up	1.613712	up	-1.50854	down	1.857134	up	1.871582	up	-2.81373	down	2.234435	up	1.871582
cfa-miR-19b	23.33125	up	2.148371	up	1.103432	up	4.097065	up	4.604815	up	-2.37276	down	1.272401	up	1.613712	up	-1.50854	down	1.857134	up	1.871582	up	-2.46201	down	2.234435	up	1.871582
cfa-miR-542	1.288372	up	12.35313	up	1.846128	up	12.58066	up	6.266817	up	5.727883	up	-3.42945	down	-35.1694	down	-1.02274	down	8.599339	up	12.09331	up	-2.18846	down	1.61804	up	1.846128
cfa-miR-8859a	-496.266	down	1.806059	up	1.07879	up	2.890354	up	-817.69	down	-29.6017	down	-817.69	down	1.148369	up	4.423567	up	28.78686	up	-129.196	down	4.339047	up	4.193812	up	1.806059
cfa-miR-7	1.109032	up	1.027102	up	1.980132	up	1.801669	up	12.09508	up	1.795825	up	1.310921	up	-8.00713	down	-2.05739	down	2.852018	up	1.722984	up	2.247569	up	3.602978	up	1.795825
cfa-miR-7	1.113605	up	1.032774	up	1.97466	up	1.808175	up	12.142	up	1.79495	up	1.316989	up	-7.96315	down	-2.05378	down	2.854836	up	1.728522	up	2.256616	up	3.612238	up	1.79495
cfa-miR-196a	10.99892	up	-32.6573	down	9.369382	up	1.007138	up	4.614198	up	1.794844	up	1.798489	up	-32.6573	down	-1.93212	down	7.913003	up	1.162817	up	1.585632	up	1.701015	up	1.794844
cfa-miR-7	1.113683	up	1.033445	up	1.976224	up	1.80697	up	12.13671	up	1.794242	up	1.315884	up	-7.9663	down	-2.05614	down	2.851034	up	1.72784	up	2.252923	up	3.607835	up	1.794242
cfa-miR-139	3.295369	up	1.717204	up	-1	down	4.698634	up	2.145016	up	-1	down	-1	down	64.49242	up	12.82248	up	1.419877	up	-1	down	-1	down	8.12936	up	1.717204
cfa-miR-374a	2.754774	up	1.093716	up	2.376623	up	7.689418	up	6.062497	up	1.64493	up	1.434985	up	-18.8408	down	3.186309	up	-18.8408	down	2.55303	up	-2.63787	down	-2.31762	down	1.64493
cfa-let-7f	-2.44479	down	-18.4461	down	2.027866	up	7.430647	up	2.832079	up	1.112415	up	-1.4666	down	2.13785	up	2.818915	up	4.51607	up	-1.23416	down	-1.36299	down	1.264262	up	1.63307
cfa-miR-454	12.35763	up	-1	down	-1	down	1.566211	up	4.290032	up	11.45347	up	1.864567	up	-1	down	-1	down	2.839753	up	1.265817	up	7.142413	up	-1	down	1.566211
cfa-miR-26a	-1.21717	down	-10.0662	down	1.04714	up	2.227795	up	3.628713	up	2.060493	up	-1.3646	down	1.514896	up	3.421588	up	1.372291	up	4.333899	up	-2.86663	down	-1.23086	down	1.514896
cfa-miR-26a	-1.21788	down	-10.072	down	1.048249	up	2.224356	up	3.627116	up	2.060544	up	-1.36459	down	1.512839	up	3.419347	up	1.373456	up	4.333142	up	-2.86829	down	-1.22875	down	1.512839
cfa-miR-92a	4.515986	up	4.298389	up	1.567676	up	-1.61554	down	-1.45072	down	1.491865	up	1.322454	up	-2.45159	down	1.455013	up	2.570794	up	1.220617	up	1.129867	up	1.807291	up	1.491865
cfa-miR-92a	4.514253	up	4.29265	up	1.558305	up	-1.63269	down	-1.45191	down	1.483837	up	1.31467	up	-2.46923	down	1.449917	up	2.566587	up	1.215422	up	1.129132	up	1.802805	up	1.483837
cfa-miR-197	1.551007	up	-1.09076	down	1.481895	up	1.12224	up	1.838338	up	2.069836	up	-1.78936	down	-1.25413	down	2.079926	up	3.391288	up	1.61244	up	-1.27217	down	1.408646	up	1.464542
cfa-miR-146b	6.742354	up	74.42123	up	3.833264	up	-1.13434	down	8.761381	up	1.441909	up	3.938628	up	-1.35169	down	1.04281	up	-1.02837	down	2.524856	up	168.9795	up	-1.2143	down	1.441909
cfa-miR-30d	1.01401	up	1.593331	up	-1.26159	down	5.234331	up	5.08388	up	1.583792	up	-1.32267	down	-1.1947	down	2.095293	up	1.423222	up	1.199043	up	2.123331	up	1.084198	up	1.423222
cfa-miR-34c	1.647685	up	-1	down	-1	down	-1	down	1.072508	up	-1	down	7.458267	up	-1	down	9.325438	up	1.419877	up	48.10105	up	18.74884	up	48.77616	up	1.419877
cfa-miR-181a	-13.7742	down	1.414108	up	3.242913	up	-1.14504	down	1.064082	up	1.166956	up	1.478801	up	2.132205	up	6.682324	up	9.811435	up	-1.31302	down	-1.89999	down	2.145034	up	1.414108
cfa-miR-181a	-13.9845	down	1.392843	up	3.196379	up	-1.16253	down	1.046475	up	1.161506	up	1.459353	up	2.084699	up	6.566133	up	9.646893	up	-1.33307	down	-1.929	down	2.112777	up	1.392843
cfa-miR-194	2.311262	up	2.148371	up	2.348331	up	1.335999	up	3.639124	up	-7.83012	down	-7.07325	down	1.173608	up	2.187549	up	-3.7154	down	1.103753	up	-4.92403	down	-3.8936	down	1.335999
cfa-miR-128	1.239201	up	1.884618	up	1.729552	up	6.618539	up	1.333182	up	-1.35981	down	-1.55733	down	-1.21232	down	1.829678	up	2.270054	up	1.357508	up	-4.91508	down	1.00364	up	1.333182
cfa-miR-101	3.488799	up	1.102048	up	1.309777	up	1.256605	up	2.705194	up	2.07335	up	-1.97711	down	2.168738	up	3.136094	up	-1.30134	down	1.313179	up	-1.48069	down	2.052206	up	1.313179
cfa-miR-130b	10.3304	up	1.74311	up	2.688556	up	5.470917	up	1.31283	up	1.327566	up	1.614358	up	-1.49815	down	-1.69024	down	2.515205	up	-1.32305	down	-1.30871	down	-50.242	down	1.31283
cfa-miR-375	8.332892	up	-1.60919	down	2.862752	up	1.544496	up	-3.81703	down	-1.39034	down	3.744906	up	-2.38039	down	1.307709	up	-4.32481	down	-1.61706	down	57.72023	up	1.201271	up	1.307709
cfa-miR-128	1.175746	up	1.803032	up	1.930338	up	6.621817	up	1.358478	up	-1.28997	down	-1.53073	down	-1.20192	down	1.7347	up	2.149977	up	1.307348	up	-5.13443	down	1.078491	up	1.293956
cfa-miR-101	3.306687	up	1.104861	up	1.187059	up	1.039695	up	2.484811	up	1.517197	up	-2.01597	down	2.24	up	3.068176	up	-1.68968	down	1.288932	up	-1.87275	down	2.030792	up	1.288932
cfa-miR-186	4.123946	up	1.133171	up	-1.04854	down	2.33486	up	1.672884	up	1.08958	up	1.2163	up	-2.05324	down	1.239997	up	1.017892	up	1.290719	up	1.124242	up	1.630106	up	1.2163
cfa-miR-425	46.92656	up	8.2443	up	2.052539	up	4.439837	up	-1.68032	down	-1.80948	down	-1.0844	down	-1.00423	down	1.209279	up	7.433428	up	-3.63838	down	1.981625	up	-2.78114	down	1.209279
cfa-miR-185	-3.04259	down	-3.15352	down	1.356028	up	9.159637	up	7.018761	up	2.490636	up	-1.33559	down	1.208919	up	2.864368	up	-1.84599	down	1.307682	up	-1.08604	down	-1.41693	down	1.208919
cfa-miR-500	4.315624	up	-2.52351	down	-4.64524	down	5.90034	up	5.067552	up	3.42046	up	1.204785	up	-4.79943	down	1.096177	up	-61.0388	down	2.059367	up	2.008784	up	-1.56015	down	1.204785
cfa-miR-8859b	-170.823	down	-1.24347	down	-1.17988	down	1.572419	up	-170.823	down	-36.221	down	-170.823	down	3.186434	up	7.625717	up	9.409161	up	-170.823	down	1.902436	up	2.688801	up	1.176123
cfa-miR-212	-1	down	-1	down	6.716613	up	10.96348	up	4.290032	up	-1	down	-1	down	-1	down	1.16568	up	4.259631	up	8.860719	up	7.142413	up	-1	down	1.16568
cfa-miR-1343	1.193971	up	1.219788	up	2.330372	up	-2.50334	down	-2.49146	down	-1.0773	down	1.155573	up	-1.40193	down	2.075612	up	2.071324	up	-1.17526	down	1.472676	up	1.866719	up	1.155573
cfa-miR-218	-422.033	down	1.155563	up	5.442895	up	14.67929	up	1.599741	up	3.874464	up	1.829076	up	-3.08675	down	-1.97841	down	1.248183	up	-30.3098	down	-29.5442	down	1.855609	up	1.155563
cfa-miR-218	-427.057	down	1.154031	up	5.303716	up	14.5011	up	1.58092	up	3.841502	up	1.846852	up	-3.1235	down	-2.00196	down	1.266745	up	-30.6706	down	-29.8959	down	1.875022	up	1.154031
cfa-miR-148a	1.146588	up	-1.2733	down	1.470574	up	9.870854	up	1.40321	up	-1.07954	down	-2.57493	down	1.219967	up	-1.77732	down	1.782371	up	1.480392	up	-8.04057	down	2.984446	up	1.146588
cfa-miR-339	2.39813	up	-3.34378	down	-1.14591	down	2.766633	up	5.323364	up	-1.21751	down	-1.26803	down	-40.1936	down	1.058558	up	2.366836	up	1.133747	up	-1.28628	down	1.21353	up	1.133747
cfa-let-7b	-18.8291	down	-40.8375	down	1.866528	up	1.826658	up	-1.40999	down	-3.78872	down	-3.39682	down	3.035519	up	1.113821	up	1.974506	up	-3.79962	down	1.575095	up	5.100324	up	1.113821
cfa-miR-320	-5.41867	down	-8.81986	down	-1.06752	down	1.119309	up	-1.09911	down	-1.67785	down	1.107411	up	1.421235	up	2.557047	up	3.126107	up	-1.14638	down	2.083636	up	1.346344	up	1.107411
cfa-miR-134	1.093164	up	-3.76815	down	-3.76815	down	-3.76815	down	2.134682	up	1.072781	up	1.731878	up	2.053815	up	3.86688	up	18.84049	up	5.710725	up	-1.05515	down	-3.76815	down	1.093164
cfa-miR-138a	3026.796	up	3.434408	up	-1	down	7.04795	up	3.217524	up	-1	down	-1	down	-1	down	-1	down	176.0647	up	8.860719	up	1.785603	up	-1	down	1.09089
cfa-miR-1838	3.148313	up	10.80045	up	2.257792	up	2.805599	up	1.366197	up	-2.07146	down	-1.2248	down	-12.5605	down	-1.53932	down	12.20863	up	-2.48071	down	1.350521	up	1.078693	up	1.078693
cfa-miR-138b	2751.633	up	3.434408	up	-1	down	2.349317	up	1.072508	up	-1	down	-1	down	-1	down	-1	down	174.6449	up	6.329084	up	1.785603	up	-1	down	1.072508
cfa-miR-98	-2.19546	down	-21.5446	down	1.070948	up	6.794629	up	12.27311	up	1.880677	up	-1.0223	down	1.166364	up	1.778764	up	-1.68598	down	1.045101	up	-1.15691	down	-1.12076	down	1.070948
cfa-miR-8884	2.030915	up	1.070656	up	-1.86259	down	11.0346	up	1.939217	up	1.602708	up	-1.25646	down	-1.09223	down	-1.75362	down	3.200612	up	3.205454	up	-5.07695	down	-1.28242	down	1.070656
cfa-miR-200c	2.213658	up	-10.0484	down	1.708198	up	3.818731	up	1.067341	up	-10.0484	down	-2.69457	down	-3.89519	down	1.566086	up	-1.17949	down	-3.96914	down	846.9183	up	-3.70819	down	1.067341
cfa-miR-6529	-1.9581	down	2.78647	up	2.040994	up	3.169683	up	-2.28983	down	-1.1203	down	1.059524	up	2.351696	up	1.214968	up	-3.21903	down	-5.65171	down	1.399896	up	-1.02918	down	1.059524
cfa-miR-660	9.898108	up	-1.14942	down	-2.64481	down	12.63936	up	9.470228	up	1.511646	up	-1.05858	down	-1.7853	down	5.019913	up	-9.73082	down	7.512506	up	-2.21079	down	-3.39918	down	1.024025
cfa-miR-193a	-6.74835	down	-3.21125	down	2.266595	up	1.01371	up	-1.23398	down	-1.59545	down	1.021258	up	2.533038	up	6.807424	up	-4.4231	down	-2.65264	down	3.463177	up	1.993584	up	1.021258
cfa-let-7a	-5.56969	down	-23.5907	down	1.445066	up	2.486498	up	-1.50965	down	-1.87293	down	-1.26339	down	2.104621	up	1.830796	up	1.982724	up	-3.63178	down	1.122969	up	1.01568	up	1.01568
cfa-miR-30e	1.053695	up	-1.54766	down	-1.46082	down	1.000329	up	-1.75226	down	1.148942	up	-1.48926	down	2.087143	up	1.424079	up	1.320745	up	-1.52398	down	-2.85426	down	-1.30209	down	1.000329

cfa-miR-448	-1	down	-1	down	-1	down	-1	down	2.68127	up	40.42402	up	3.729133	up	-1	down	-1	down	-1	down	-1	down	-1	down	-1	down	-1
cfa-miR-429	-1	down	-1	down	-1	down	-1	down	-1	down	-1	down	-1	down	-1	down	-1	down	-1	down	-1	down	311.5878	up	1.354893	up	-1
cfa-miR-216b	-1	down	-1	down	-1	down	-1	down	-1	down	4.716135	up	17.71338	up	-1	down	-1	down	156.1864	up	1.265817	up	-1	down	-1	down	-1
cfa-miR-31	-1	down	-1	down	2.985161	up	-1	down	-1	down	-1	down	-1	down	-1	down	-1	down	15.61865	up	18.98725	up	-1	down	-1	down	-1
cfa-miR-491	-1	down	-1	down	-1	down	-1	down	-1	down	-1	down	-1	down	-1	down	-1	down	-1	down	-1	down	-1	down	-1	down	-1
cfa-miR-150	-1	down	-1	down	-1	down	-1	down	8.580064	up	-1	down	9.322833	up	-1	down	14.571	up	-1	down	7.594901	up	-1	down	-1	down	-1
cfa-miR-135a-5p	-1	down	6.868817	up	-1	down	-1	down	-1	down	-1	down	-1	down	-1	down	-1	down	-1	down	-1	down	-1	down	-1	down	-1
cfa-miR-124	-1	down	-1	down	-1	down	-1	down	1.608762	up	-1	down	-1	down	-1	down	-1	down	-1	down	-1	down	-1	down	-1	down	-1
cfa-miR-124	-1	down	-1	down	-1	down	-1	down	1.608762	up	-1	down	-1	down	-1	down	-1	down	-1	down	-1	down	-1	down	-1	down	-1
cfa-miR-124	-1	down	-1	down	-1	down	-1	down	1.608762	up	-1	down	-1	down	-1	down	-1	down	-1	down	-1	down	-1	down	-1	down	-1
cfa-miR-34a	-1	down	-1	down	-1	down	2.349317	up	13.9426	up	-1	down	-1	down	-1	down	15.15384	up	1.419877	up	8.860719	up	-1	down	-1	down	-1
cfa-miR-497	-1	down	-1	down	-1	down	2.349317	up	1.072508	up	6.063603	up	-1	down	-1	down	2.914199	up	-1	down	2.531634	up	7.142413	up	1.354893	up	-1
cfa-miR-136	-1	down	-1	down	-1	down	-1	down	-1	down	-1	down	-1	down	15.47818	up	-1	down	-1	down	-1	down	-1	down	-1	down	-1
cfa-miR-380	-1	down	-1	down	-1	down	-1	down	-1	down	-1	down	-1	down	-1	down	-1	down	7.099383	up	-1	down	-1	down	-1	down	-1
cfa-miR-376a	-1	down	-1	down	-1	down	-1	down	1.072508	up	-1	down	-1	down	-1	down	-1	down	2.839753	up	1.265817	up	-1	down	-1	down	-1
cfa-miR-376a	-1	down	-1	down	-1	down	-1	down	1.072508	up	-1	down	-1	down	-1	down	-1	down	2.839753	up	1.265817	up	-1	down	-1	down	-1
cfa-miR-376a	-1	down	-1	down	-1	down	-1	down	1.072508	up	-1	down	-1	down	-1	down	-1	down	2.839753	up	1.265817	up	-1	down	-1	down	-1
cfa-miR-487b	-1	down	-1	down	-1	down	-1	down	1.072508	up	-1	down	-1	down	-1	down	4.079879	up	-1	down	-1	down	-1	down	-1	down	-1
cfa-miR-219-3p	-1	down	-1	down	-1	down	2.349317	up	-1	down	7.41107	up	2.79685	up	-1	down	4.662719	up	-1	down	2.531634	up	-1	down	-1	down	-1
cfa-miR-144	-1	down	-1	down	-1	down	3.132422	up	3.753778	up	-1	down	-1	down	30.95635	up	57.70115	up	-1	down	1.265817	up	-1	down	-1	down	-1
cfa-miR-338	-1	down	-1	down	-1	down	-1	down	-1	down	-1	down	-1	down	-1	down	-1	down	38.33667	up	-1	down	-1	down	-1	down	-1
cfa-miR-135a-5p	-1	down	5.151613	up	-1	down	-1	down	-1	down	-1	down	-1	down	-1	down	-1	down	-1	down	-1	down	-1	down	-1	down	-1
cfa-miR-153	-1	down	-1	down	-1	down	75.17813	up	-1	down	-1	down	-1	down	-1	down	2.914199	up	-1	down	3.797451	up	-1	down	-1	down	-1
cfa-miR-205	-1	down	-1	down	-1	down	1.566211	up	3.753778	up	-1	down	-1	down	-1	down	17.48519	up	-1	down	2.531634	up	421.4023	up	-1	down	-1
cfa-miR-210	-1	down	-1	down	-1	down	-1	down	-1	down	-1	down	-1	down	20.63757	up	2.914199	up	1.419877	up	-1	down	-1	down	4.06468	up	-1
cfa-miR-223	-1	down	-1	down	-1	down	14.87901	up	17.69638	up	-1	down	-1	down	-1	down	6.411238	up	-1	down	7.594901	up	-1	down	-1	down	-1
cfa-miR-489	-1	down	-1	down	-1	down	-1	down	-1	down	-1	down	-1	down	-1	down	-1	down	-1	down	-1	down	-1	down	1.354893	up	-1
cfa-miR-200b	-1	down	-1	down	-1	down	-1	down	-1	down	-1	down	-1	down	-1	down	8.159758	up	-1	down	-1	down	624.9611	up	-1	down	-1
cfa-miR-200a	-1	down	-1	down	3.731451	up	3.915528	up	-1	down	-1	down	-1	down	-1	down	10.49112	up	-1	down	-1	down	1431.161	up	-1	down	-1
cfa-miR-208a	-1	down	-1	down	-1	down	5.481739	up	-1	down	-1	down	-1	down	-1	down	5.245558	up	-1	down	-1	down	-1	down	-1	down	-1
cfa-miR-190a	-1	down	-1	down	-1	down	-1	down	-1	down	-1	down	-1	down	-1	down	1.74852	up	-1	down	-1	down	-1	down	-1	down	-1
cfa-miR-490	-1	down	-1	down	-1	down	-1	down	-1	down	-1	down	-1	down	-1	down	4.662719	up	-1	down	-1	down	-1	down	-1	down	-1
cfa-miR-211	-1	down	-1	down	-1	down	-1	down	1.608762	up	-1	down	-1	down	-1	down	-1	down	-1	down	1.265817	up	-1	down	-1	down	-1
cfa-miR-299	-1	down	-1	down	-1	down	-1	down	-1	down	-1	down	-1	down	-1	down	2.331359	up	-1	down	-1	down	-1	down	-1	down	-1
cfa-miR-346	-1	down	1.717204	up	-1	down	-1	down	-1	down	-1	down	-1	down	-1	down	-1	down	26.97766	up	-1	down	-1	down	-1	down	-1
cfa-miR-452	-1	down	-1	down	-1	down	-1	down	1.072508	up	-1	down	-1	down	-1	down	46.62719	up	-1	down	-1	down	13.39202	up	-1	down	-1
cfa-miR-483	-1	down	1.717204	up	7.462903	up	-1	down	-1	down	-1	down	-1	down	-1	down	-1	down	-1	down	-1	down	2.678405	up	-1	down	-1
cfa-miR-487a	-1	down	-1	down	-1	down	-1	down	-1	down	-1	down	-1	down	-1	down	1.74852	up	-1	down	-1	down	-1	down	-1	down	-1
cfa-miR-504	-1	down	-1	down	-1	down	-1	down	5.898794	up	-1	down	-1	down	10.31879	up	32.63903	up	-1	down	2.531634	up	-1	down	-1	down	-1
cfa-miR-551a	-1	down	-1	down	-1	down	-1	down	-1	down	-1	down	-1	down	-1	down	2.331359	up	-1	down	-1	down	-1	down	-1	down	-1
cfa-miR-885	-1	down	-1	down	-1	down	2.349317	up	4.290032	up	-1	down	-1	down	18.05788	up	10.49112	up	-1	down	-1	down	-1	down	-1	down	-1
cfa-miR-8803	-1	down	-1	down	-1	down	-1	down	-1	down	-1	down	-1	down	-1	down	1.16568	up	-1	down	-1	down	-1	down	-1	down	-1
cfa-miR-8829	-1	down	-1	down	5.224032	up	2.349317	up	2.145016	up	-1	down	-1	down	-1	down	-1	down	1.419877	up	1.265817	up	-1	down	-1	down	-1
cfa-miR-8835	-1	down	3.434408	up	-1	down	-1	down	-1	down	-1	down	-1	down	-1	down	-1	down	-1	down	-1	down	-1	down	-1	down	-1
cfa-miR-2387	-1	down	-1	down	-1	down	1.566211	up	1.608762	up	4.042402	up	7.458267	up	-1	down	-1	down	2.839753	up	3.797451	up	-1	down	13.54893	up	-1
cfa-miR-8876	-1	down	-1	down	-1	down	-1	down	2.145016	up	-1	down	-1	down	-1	down	-1	down	-1	down	1.265817	up	-1	down	-1	down	-1
cfa-miR-1185	-1	down	-1	down	-1	down	-1	down	1.072508	up	-1	down	-1	down	-1	down	1.74852	up	-1	down	2.531634	up	-1	down	-1	down	-1
cfa-miR-6516	-1	down	-1	down	-1	down	-1	down	-1	down	-1	down	-1	down	-1	down	2.331359	up	-1	down	-1	down	-1	down	-1	down	-1
cfa-miR-8908b	-1	down	8.586021	up	2.238871	up	-1	down	29.49397	up	-1	down	-1	down	-1	down	1.16568	up	35.49692	up	-1	down	-1	down	-1	down	-1
cfa-miR-8908f	-1	down	5.151613	up	-1	down	-1	down	13.9426	up	-1	down	-1	down	-1	down	-1	down	14.19877	up	1.265817	up	-1	down	-1	down	-1
cfa-miR-2483	-1	down	-1	down	-1	down	-1	down	-1	down	-1	down	2.79685	up	-1	down	-1	down	-1	down	-1	down	-1	down	-1	down	-1
cfa-miR-8908e	-1	down	15.45484	up	7.462903	up	-1	down	23.05892	up	-1	down	1.864567	up	-1	down	-1	down	29.81741	up	-1	down	-1	down	-1	down	-1
cfa-miR-449a	1.647685	up	-1	down	-1	down	-1	down	5.898794	up	-1	down	-1	down	-1	down	-1	down	-1	down	-1	down	-1	down	-1	down	-1
cfa-miR-33a	3.295369	up	1.717204	up	-1	down	7.04795	up	5.362541	up	5.389869	up	-1	down	-1	down	-1	down	-1	down	1.265817	up	-1	down	-1	down	-1
cfa-miR-1835	27.18679	up	10.30323	up	11.94065	up	99.45441	up	1.072508	up	-1	down	-1	down	-1	down	-1	down	-1	down	-1	down	-1	down	-1	down	-1
cfa-miR-371	50.25438	up	-1	down	17.91097	up	-1	down	87.94566	up	-1	down	-1	down	-1	down	-1	down	-1	down	-1	down	88.38737	up	100.2621	up	-1
cfa-miR-371	50.25438	up	-1	down	17.91097	up	-1	down	87.94566	up	-1	down	-1	down	-1	down	-1	down	-1	down	-1	down	88.38737	up	100.2621	up	-1
cfa-miR-17	5.766895	up	-1	down	-1	down	3.915528	up	-1	down	6.737337	up	1.864567	up	-1	down	-1	down	-1	down	-1	down	-1	down	-1	down	-1
cfa-miR-350	6.590737	up	8.586021	up	-1	down	-1	down	6.971302	up	-1	down	-1	down	-1	down	-1	down	14.19877	up	17.72144	up	-1	down	10.83915	up	-1
cfa-miR-18b	2.471527	up	-1	down	-1	down	-1	down	-1	down	-1	down	-1	down	-1	down	-1	down	-1	down	1.265817	up	-1	down	1.354893	up	-1
cfa-miR-202	3.295369	up	-1	down	-1	down	-1	down	6.435049	up	-1	down	-1	down	-1	down	-1	down	-1	down	6.329084	up	-1	down	-1	down	-1
cfa-miR-301a	8.238422	up	-1	down	-1	down	2.349317	up	-1	down	-1	down	-1	down	-1	down	-1	down	8.519261	up	-1	down	1.785603	up	-1	down	-1
cfa-miR-301b	74.96965	up	-1	down	2.985161	up	15.66211	up	1.608762	up	-1	down	3.729133	up	-1	down	-1	down	-1	down	2.531634	up	-1	down	-1	down	-1
cfa-miR-326	5.766895	up	-1	down	-1	down	16.44522	up	2.68127	up	-1	down	-1	down	-1	down	2.914199	up	8.519261	up	-1	down	-1	down	-1	down	-1
cfa-miR-208b	-1.40735	down	3.531791	up	2.673702	up	2.234088	up	-1.56151	down	-2.23718	down	-5.38914	down	4.792235	up	5.142951	up	1.978251	up	-1.98457	down	-15.0726	down	-1.01133	down	-1.01133
cfa-let-7a	-5.59523	down	-24.0389	down	1.418429	up	2.487965	up	-1.55525	down	-1.87424	down	-1.26563	down	2.060131	up	1.813497	up	1.947089	up	-3.68755	down	1.119929	up	-1.02089	down	-1.02089
cfa-miR-125a	-1802.43	down	-131.204	down	-1.24816	down	-22.1312	down	1.692276	up	1.536656	up	-1.03499	down	3.974526	up	6.856909	up	-2.65571	down	-8.1835	down	-1.02584	down	1.572561	up	-1.03499
cfa-miR-130a	-26.3771	down	-26.3771	down	-26.3771	down	-3.74252	down	-1.04655	down	-2.44691	down	1.590501	up	-1.1361	down	2.452706	up	3.929586	up	3.215282	up	-1.73789	down	1.797821	up	-1.04655
cfa-miR-323	-1.65168	down	1.68264	up	-16.3287	down	-1.09743	down	-1.79115	down	-3.02951	down	2.740554	up	2.527772	up	7.924129	up	6.695617	up	1.240338	up	-3.65785	down	-16.3287	down	-1.09743
cfa-miR-21	1.042146	up	-4.37756	down	-4.98385	down	-1.16073	down	1.401253	up	-1.7728	down	-2.16878	down	1.038483	up	3.257131	up	2.197551	up	2.46821	up	-1.90295	down	-1.10908	down	-1.10908
cfa-miR-424	-3.43041	down	4.860961	up	2.145563	up	1.350844	up	-1.05402	down	1.579371	up	-8.08371	down	-22.6089	down	-2.15505	down	5.840551	up	5.038875	up	-3.16545	down	-1.11246	down	-1.11246
cfa-miR-222	-3.84768	down	-4.38694	down	-3.8655	down	-1.31555	down	1.664828	up	1.281858	up	1.299433	up	-1.89333	down	1.844955	up	1.208469	up	-1.11849	down	-1.12886	down	-4.67202	down	-1.11849
cfa-miR-125b	-424.354	down	-9.6436	down	-4.44264	down	-5.53424	down	-1.22237	down	1.798889	up	1.325947	up	3.879702	up	7.141181	up	-1.17558	down	-1.16153	down	1.832475	up	1.096779	up	-1.17558
cfa-miR-29a	10.91238	up	-2.52852	down	-5.23362	down	-4.23364	down	2.771639	up	-1.45931	down	1.277383	up	-1.17581	down	4.043359	up	1.537118	up	-1.80681	down	-1.09369	down	-1.02155	down	-1.17581

cfa-miR-125b	-424.523	down	-9.64745	down	-4.43974	down	-5.55174	down	-1.22516	down	1.794961	up	1.320382	up	3.868319	up	7.13407	up	-1.18549	down	-1.16417	down	1.831744	up	1.096341	up	-1.18549
cfa-miR-676	-8.79236	down	-1.28004	down	-1.68306	down	-2.24551	down	-1.09306	down	-1.08752	down	-1.57183	down	4.107629	up	1.126919	up	-8.79236	down	-6.946	down	1.827772	up	-1.29787	down	-1.28004
cfa-miR-142	182.3125	up	17.31717	up	20.72119	up	61.51535	up	33.74577	up	-1.39824	down	-5.38914	down	-1.2984	down	5.181618	up	-1.93008	down	-2.38148	down	-16.8824	down	-22.2492	down	-1.2984
cfa-miR-30a	-37.3309	down	-36.905	down	-1.31997	down	-3.86477	down	21.20091	up	-1.02209	down	-2.5064	down	1.073165	up	1.963678	up	1.304915	up	3.761148	up	-7.63006	down	-1.31141	down	-1.31141
cfa-miR-22	-7.815	down	-4.36337	down	-3.20212	down	-3.45604	down	1.155704	up	-1.90969	down	-1.32353	down	3.311694	up	5.660427	up	-1.30692	down	1.396732	up	-1.50969	down	-1.03711	down	-1.32353
cfa-let-7d	-39.3353	down	-8.3873	down	1.939068	up	-1.33704	down	1.300667	up	-2.38706	down	-2.03871	down	3.570295	up	3.724794	up	-2.14302	down	-4.33914	down	-2.14142	down	1.072422	up	-1.33704
cfa-miR-433	-5.02421	down	-1.4629	down	5.64448	up	2.182132	up	-1.33844	down	-2.48575	down	2.041142	up	6.674899	up	17.40096	up	-1.76924	down	1.259719	up	-5.02421	down	-5.02421	down	-1.33844
cfa-miR-574	-3.95273	down	-87.9236	down	-11.7814	down	1.131146	up	6.038098	up	3.019111	up	-3.25207	down	-1.48187	down	2.346643	up	12.35398	up	3.440831	up	-9.84806	down	3.359357	up	-1.3433
cfa-miR-99a	-176.094	down	-8.57452	down	-2.07122	down	1.395822	up	2.84989	up	1.213822	up	1.207609	up	1.304233	up	1.114213	up	-2.88119	down	1.221292	up	-7.25503	down	-2.74248	down	-1.35298
cfa-miR-99a	-176.102	down	-8.57491	down	-2.07131	down	1.395778	up	2.849807	up	1.213767	up	1.207688	up	1.304174	up	1.114219	up	-2.88132	down	1.221237	up	-7.25535	down	-2.7426	down	-1.35303
cfa-miR-331	-1.37216	down	-11.3045	down	-1.08197	down	-2.0622	down	1.280809	up	-2.79647	down	-1.10232	down	1.597411	up	-2.15505	down	-7.96158	down	-2.23264	down	-11.3045	down	1.078693	up	-1.37216
cfa-miR-543	-12.5605	down	1.50386	up	1.663637	up	1.558666	up	-1.95189	down	-3.10719	down	1.484467	up	5.5453	up	14.70961	up	2.826073	up	-1.98457	down	-12.5605	down	-12.5605	down	-1.38168
cfa-miR-423a	1.802966	up	3.434754	up	-1.75386	down	-4.13419	down	-4.31115	down	-5.16223	down	-1.39987	down	3.157926	up	-1.68011	down	4.707063	up	-8.0946	down	1.445518	up	1.373634	up	-1.39987
cfa-miR-505	2.172664	up	-1.30036	down	-6.73224	down	-20.0968	down	-3.74763	down	-1.86431	down	-1.0265	down	1.026907	up	-6.89617	down	2.684769	up	-3.17531	down	1.732576	up	1.61804	up	-1.42042
cfa-miR-149	-2.17804	down	-37.6815	down	-37.6815	down	-12.0295	down	-1.43404	down	1.394615	up	-1.44352	down	1.300749	up	4.222632	up	4.521716	up	4.064693	up	-1.45538	down	3.02034	up	-1.43404
cfa-miR-206	-2.64269	down	1.577475	up	2.102398	up	1.582645	up	-1.44998	down	-5.3858	down	-3.18449	down	2.527772	up	3.390956	up	-2.30001	down	-3.22493	down	-12.1928	down	1.327622	up	-1.44998
cfa-miR-106b	2.295645	up	-2.43817	down	-2.24408	down	2.743252	up	3.650307	up	1.233698	up	-25.121	down	-25.121	down	-1.39036	down	-1.47437	down	1.058164	up	-4.01961	down	-4.63524	down	-1.47437
cfa-miR-874	-8.79236	down	-8.79236	down	-8.79236	down	-2.24551	down	-4.09897	down	-1.00386	down	-8.79236	down	-8.79236	down	1.988681	up	2.099368	up	4.175067	up	4.366345	up	-1.62233	down	-1.62233
cfa-miR-193b	-91.6917	down	2.116266	up	-8.77595	down	-1.69692	down	1.976774	up	-3.40237	down	1.453964	up	4.642184	up	1.875172	up	7.587813	up	-1.36673	down	-4.10804	down	-1.99043	down	-1.69692
cfa-miR-10b	-31.4013	down	-9.14314	down	1.354675	up	-1.08374	down	-1.77445	down	-1.79261	down	-2.59093	down	-31.4013	down	-1.38144	down	1.944337	up	-12.4036	down	-31.4013	down	-2.89703	down	-1.79261
cfa-miR-129	-4.01947	down	-5.6277	down	-24.4044	down	-14.7484	down	-1.93224	down	-10.0407	down	-11.0419	down	3.116134	up	6.491437	up	46.71042	up	1.633291	up	1.595059	up	21.78273	up	-1.93224
cfa-miR-129	-4.01947	down	-5.6277	down	-24.4044	down	-14.7484	down	-1.94074	down	-10.0407	down	-11.0419	down	3.116134	up	6.491437	up	46.68643	up	1.630618	up	1.595059	up	21.78273	up	-1.94074
cfa-miR-147	-13.8166	down	1.864289	up	-13.8166	down	1.757042	up	13.81722	up	-13.8166	down	-4.94004	down	-13.8166	down	-4.74112	down	-1.94616	down	-1.21279	down	196.0516	up	-10.1975	down	-1.94616
cfa-let-7e	-459.522	down	-110.23	down	-3.1904	down	-24.6646	down	-1.78543	down	-4.41749	down	-2.0488	down	2.41497	up	-1.18701	down	2.049325	up	-5.32162	down	-1.14479	down	-1.23743	down	-2.0488
cfa-miR-127	-20.2013	down	-3.52427	down	-2.54863	down	-1.62852	down	-1.63773	down	-4.57448	down	-1.81234	down	1.216788	up	4.493168	up	-9.76768	down	-3.75651	down	-5.64878	down	-1.9973	down	-2.08192
cfa-miR-29c	3.716759	up	-3.76815	down	3.168832	up	7.481597	up	4.980925	up	-3.76815	down	-3.76815	down	-3.76815	down	1.546752	up	1.884048	up	-2.97685	down	-2.1103	down	-3.76815	down	-2.1103
cfa-miR-29c	3.716759	up	-3.76815	down	3.168832	up	7.481597	up	4.980925	up	-3.76815	down	-3.76815	down	-3.76815	down	1.546752	up	1.884048	up	-2.97685	down	-2.1103	down	-3.76815	down	-2.1103
cfa-miR-135b	-13.8166	down	-13.8166	down	-13.8166	down	-13.8166	down	1.203185	up	-2.05075	down	-2.11716	down	-13.8166	down	-11.8528	down	1.541494	up	1.191007	up	-3.86888	down	-1.69959	down	-2.11716
cfa-miR-122	-13.1541	down	-1.22843	down	-1.35161	down	-1.69446	down	-3.55354	down	-8.50023	down	-3.65552	down	1.72644	up	2.481152	up	-4.68164	down	-16.0452	down	-1.4902	down	-1.75301	down	-2.15104
cfa-miR-365	-5.89522	down	5.091437	up	-19.5235	down	-3.32244	down	5.645867	up	-1.01056	down	-4.11277	down	-2.35334	down	1.464081	up	-7.32971	down	-2.21356	down	2.132401	up	1.580843	up	-2.21356
cfa-miR-365	-5.89522	down	5.091437	up	-19.5235	down	-3.32244	down	5.645867	up	-1.01056	down	-4.11277	down	-2.35334	down	1.464081	up	-7.32971	down	-2.21356	down	2.132401	up	1.580843	up	-2.21356
cfa-miR-486	-18.4018	down	-4.59012	down	-1.03463	down	-1.23963	down	-5.35906	down	-2.27314	down	-1.8653	down	2.668655	up	5.268246	up	-7.26143	down	-5.95715	down	-4.02426	down	-2.32198	down	-2.27314
cfa-miR-628	-1.08902	down	-6.28026	down	-2.8051	down	4.114877	up	-1.17113	down	1.716449	up	-6.28026	down	-6.28026	down	-1.79588	down	-2.21155	down	-6.28026	down	-6.28026	down	-2.31762	down	-2.31762
cfa-miR-889	-1.69403	down	-2.43817	down	-12.5605	down	-12.5605	down	-1.95189	down	-12.5605	down	-2.24547	down	-2.43449	down	1.856102	up	11.41733	up	3.627989	up	-12.5605	down	-12.5605	down	-2.43449
cfa-miR-432	-34.7615	down	-4.63252	down	-1.2459	down	-1.15727	down	-4.45031	down	-2.56062	down	-1.82846	down	1.855641	up	3.907585	up	-20.1693	down	-28.2801	down	-2.71835	down	-3.9142	down	-2.56062
cfa-miR-379	-17.0919	down	-7.78996	down	-3.22966	down	-1.95222	down	-2.55848	down	-2.79647	down	-5.51869	down	1.359567	up	2.265663	up	2.802189	up	-3.52261	down	-19.9775	down	-5.80769	down	-2.79647
cfa-miR-1843	-23.865	down	-3.47439	down	-3.19781	down	1.476631	up	-1.27152	down	-5.90366	down	-2.84427	down	-23.865	down	-2.40859	down	-1.29291	down	-2.09482	down	-6.68261	down	-23.865	down	-2.84427
cfa-miR-451	-22.7	down	-7.17179	down	-2.63265	down	-1.05357	down	-1.7901	down	-2.84964	down	-5.88705	down	-1.42871	down	1.606775	up	-13.171	down	-5.69855	down	-12.8536	down	-2.72024	down	-2.84964
cfa-miR-769	-1.52463	down	-2.92581	down	-5.02421	down	-5.02421	down	1.707746	up	-5.02421	down	-5.02421	down	-5.02421	down	-2.87341	down	4.521715	up	3.527212	up	1.421601	up	-3.70819	down	-2.87341
cfa-miR-615	-32.6573	down	-32.6573	down	-32.6573	down	-32.6573	down	-3.58229	down	-1.31006	down	-2.91912	down	-2.53187	down	-9.33857	down	-1.35295	down	-4.2999	down	-2.28616	down	1.286134	up	-2.91912
cfa-miR-126	-3.6591	down	-2.92581	down	-1.83607	down	6.962041	up	-3.5134	down	-15.0726	down	-15.0726	down	2.396117	up	2.784154	up	-1.76924	down	-2.97685	down	-15.0726	down	-5.56229	down	-2.92581
cfa-miR-29b	5.418294	up	-8.41169	down	-7.03825	down	-2.23579	down	1.373622	up	-1.75017	down	-1.34728	down	-57.7783	down	-2.25301	down	1.007556	up	-5.07168	down	-3.59532	down	-7.10737	down	-3.17787
cfa-miR-29b	5.518103	up	-8.41169	down	-7.03825	down	-2.23579	down	1.382903	up	-1.75017	down	-1.34728	down	-57.7783	down	-2.25301	down	1.007556	up	-5.07168	down	-3.59532	down	-7.10737	down	-3.17787
cfa-miR-10a	-44.8356	down	1.586372	up	21.95073	up	15.92065	up	1.900882	up	-15.7603	down	-19.3069	down	-1.45741	down	-3.30911	down	-4.981	down	-72.4368	down	-27.4516	down	-9.30798	down	-3.30911
cfa-miR-671	-5.91762	down	-4.65284	down	1.372526	up	-7.49595	down	-3.77732	down	1.321065	up	-1.10981	down	-1.23889	down	-2.46754	down	-1.17778	down	-4.13152	down	-5.46054	down	-1.96569	down	-3.44572
cfa-miR-363	30.89555	up	28.79549	up	-1.9235	down	-4.52876	down	-3.03862	down	-3.72863	down	1.979289	up	-60.2905	down	-7.95712	down	-3.86016	down	12.0513	up	-60.2905	down	-7.41638	down	-3.86016
cfa-miR-195	-36.4255	down	-36.4255	down	-4.88087	down	-7.75236	down	-4.24536	down	-1.50181	down	-1.86054	down	1.274782	up	-1.04161	down	-36.4255	down	-4.1109	down	-1.23634	down	-1.22202	down	-4.24536
cfa-miR-20b	12.79002	up	15.0386	up	-3.36612	down	-4.27716	down	-2.08202	down	-10.0484	down	-5.38914	down	-10.0484	down	-10.0484	down	-10.0484	down	6.928453	up	-10.0484	down	-10.0484	down	-4.27716
cfa-miR-362	2.529895	up	-8.79236	down	-8.79236	down	4.007998	up	1.768737	up	-4.35007	down	-1.34728	down	-8.79236	down	-1.67615	down	-8.79236	down	2.303486	up	-8.79236	down	-8.79236	down	-4.35007
cfa-miR-1836	-3.90685	down	2.684272	up	1.231787	up	-4.38409	down	-1.12319	down	-19.1092	down	-4.41909	down	-1.5356	down	-7.68323	down	-4.83592	down	-4.06837	down	-2.35435	down	-25.3393	down	-4.38409
cfa-miR-1468	-196.423	down	-4.21949	down	-2.00155	down	-3.47295	down	-10.2874	down	-5.14684	down	-4.03663	down	2.662254	up	2.227684	up	-14.5492	down	-69.7306	down	-13.426	down	-4.84195	down	-4.42802
cfa-miR-381	-11.4891	down	-9.64601	down	-4.9323	down	-2.75147	down	-1.42426	down	-5.54043	down	-14.2139	down	-1.36981	down	1.579006	up	2.368007	up	1.160613	up	-16.4916	down	-8.50466	down	-4.9323
cfa-miR-196b	-69.8787	down	-5.2934	down	-345.414	down	-345.414	down	-5.23678	down	1.320495	up	-1.10598	down	-1.53905	down	1.111974	up	-345.414	down	-90.9595	down	-11.3791	down	-12.1399	down	-5.2934
cfa-let-7c	-77.0006	down	-14.6172	down	-1.3243	down	-1.05414	down	-16.0946	down	1.369289	up	1.337703	up	2.431083	up	-1.19194	down	-1.98366	down	-6.0116	down	-19.7619	down	-11.4577	down	-6.0116
cfa-miR-411	-13.3405	down	-43.9618	down	-2.6776	down	-3.11876	down	-9.10883	down	-13.0502	down	-43.9618	down	1.114928	up	1.153435	up	-6.19234	down	-6.946	down	-43.9618	down	-6.48934	down	-6.48934
cfa-miR-18a	2.987983	up	-6.58306	down	-11.3045	down	-1.11042	down	1.09106	up	-11.3045	down	-11.3045	down	-11.3045	down	-11.3045	down	-11.3045	down	-2.23264	down	-3.16545	down	-1.66869	down	-6.58306
cfa-miR-1	-131.997	down	-65.8599	down	-19.2313	down	-5.56311	down	-18.8302	down	-11.2208	down	-17.0379	down	1.467142	up	3.444221	up	-6.59364	down	-1.32875	down	-7.14865	down	1.328839	up	-7.14865
cfa-miR-1	-131.997	down	-65.8599	down	-19.2313	down	-5.56311	down	-18.8302	down	-11.2208	down	-17.0379	down	1.467142	up	3.444221	up	-6.59364	down	-1.32875	down	-7.14865	down	1.328839	up	-7.14865
cfa-miR-485	-8.13134	down	-3.51097	down	-2.44809	down	-19.2472	down	-11.2429	down	-8.94871	down	-3.07951	down	2.096602	up	1.150397	up	3.226433	up	-23.8148	down	-16.8824	down	-14.8328	down	-8.13134
cfa-miR-217	-8.79236	down	-8.79236	down	-8.79236	down	-8.79236	down	-8.79236	down	-8.79236	down	-8.79236	down	-8.79236	down	-8.79236	down	1.130429	up	-8.79236	down	-8.79236	down	-8.79236	down	-8.79236
cfa-miR-329b	-8.79236	down	-8.79236	down	-8.79236	down	-8.79236	down	-8.79236	down	-1.86431	down	-8.79236	down	-8.79236	down	-1.88567	down	-1.23847	down	-2.31533	down	-8.79236	down	-8.79236	down	-8.79236
cfa-miR-410	-13.8166	down	-13.8166	down	-2.31421	down	-8.82165	down	-4.29416	down	-4.10149	down	-4.94004	down	-13.8166	down	-1.03068	down	-2.4327	down	-13.8166	down	-13.8166	down	-13.8166	down	-8.82165
cfa-miR-133c	-9.65597	down	-23.865	down	-23.865	down	-3.04748	down	-3.17879	down	-23.865	down	-23.865	down	-23.865	down	-1.02365	down	-5.60259	down	1.166897	up	-23.865	down	1.476106	up	-9.65597
cfa-miR-133a	-9.65597	down	-23.865	down	-23.865	down	-3.04748	down	-3.17879	down	-23.865	down	-23.865	down	-23.865	down	-1.02365	down	-5.60259	down	1.166897	up	-23.865	down	1.476106	up	-9.65597
cfa-miR-199	-105.796	down	-291.849	down	-10.2525	down	-38.2072	down	-1.49054	down	3.678105	up	-1.50368	down	1.0668	up	5.213602	up	-9.80454	down	3.2538	up	-89.8142	down	-1.40243	down	-9.80454
cfa-miR-199	-105.265	down	-290.386	down	-10.3996	down	-38.0157	down	-1.49679	down	3.652724	up	-1.51415	down	1.048895	up	5.217366	up	-9.82361	down	3.230211	up	-89.364	down	-1.421	down	-9.82361
cfa-miR-199	-105.265	down	-290.386	down	-10.3996	down	-38.0157	down	-1.49679	down	3.652724	up	-1.51415	down	1.048895	up	5.217366	up	-9.82361	down	3.230211	up	-89.364	down	-1.421	down	-9.82361
cfa-miR-204	-302.708	down	-22.035	down	-302.708	down	-77.3097	down	3.961118	up	5.577568	up	3.421665	up	-10.6675	down	-3.63194	down	-302.708	down	1.317216	up	-302.708	down	-1.18211	down	-10.6675
cfa-miR-494	-15.0726	down	-15.0726	down	-1.55359	down	1.039111	up	-1.40536	down	-15.0726	down	-15.0726	down	-1.16856	down	1.27607	up	-3.53848	down	-11.9074	down	-15.0726	down	-15.0726	down	-11.9074
cfa-miR-145	-12.5605	down	-12.5605	down	-12.5605	down	-12.5605	down	-1.56151	down	-12.5605	down	-12.5605	down	-12.5605	down	5.661112	up	-12.5605	down	1.310107	up	-2.81373	down	6.14855	up	-12.5605
cfa-miR-105a	-13.8166	down	-13.8166	down	-13.8166	down	-13.8166	down	-13.8166	down	-13.8166	down	-13.8166	down	-13.8166	down	-2.63395	down	-13.8166	down	-13.8166	down	-13.8166	down	-13.8166	down	-13.8166
cfa-miR-455	-47.7299	down	-47.7299	down	-47.7299	down	-20.3165	down	-29.6687	down	-1.33668	down	-47.7299	down	1.080955	up	11.51516	up	3.361538	up	12.86239	up	-10.6922	down	2.725119	up	-14.5844
cfa-miR-383	-30.1452	down	-30.1452	down	9.333199	up	-12.8315	down	-1.14723	down	-30.1452	down	-30.1452	down	1.540361	up	1.218067	up	-30.1452	down	-7.93828	down	-30.1452	down	-30.1452	down	-30.1452
cfa-miR-214	-121.208	down	-116.301	down	-33.4508	down	-36.4323	down	-41.3801	down	2.246767	up	-1.33055	down	1.795473	up	2.860032	up	-70.3273	down	-4.04547	down	-199.712	down	-49.1335	down	-36.4323

cfa-miR-143	-2011.18 down	-997.041 down	-564.142 down	-799.875 down	-491.192 down	-12.7445 down	-289.971 down	2.23618 up	4.860903 up	-6029.13 down	-7.88219 down	-13.6913 down	3.390577 up	-289.971
